# A Gamboge Case

**Published:** 1887-11

**Authors:** J. A. Biegler

**Affiliations:** Rochester, N. Y.


### A GAMBOGE CASE.

J. A. Biegler, M. D., Rochester, N. Y.

Wm. M., age, fourteen years, March 27th, 1886, complained of, first, pinching pain between umbilicus and region of bladder; second, heavy pain in bladder after urinating; third, urinates very seldom. The first and third of these symptoms are to be found in the provings of this remedy; the pinching pain is given as anywhere or in entire abdomen. THE SECOND SYMPTOM IS NOT TO BE FOUND IN THE PROVING. On the above date I gave two doses of the CM potency, one at once, and the other in four days. April 28th, a month later, the prescription was repeated, with the result of a complete cure. The chief point in this case, and the object of its publication, is the fact that the second symptom disappeared under this remedy at the same time with the others. There were but the three symptoms in the case, but the previous history may throw a ray of light upon these symptoms.

The boy had been dosed without stint during three years with pumpkin-seed tea for tapeworm. After this, and about three months prior to this case, he was brought to me for a gastric difficulty, accompanied by a deep jaundiced state of the body, from which he recovered, and then followed the three symptoms cured with Gamboge, since which he has been perfectly well, and remains so now.—*Clinical Bureau, I. H. A.*

